# POLICY SERIES: INEQUITABLE IMPACTS OF POLICY AND PAYMENT CHANGES ON CARE USE AND OUTCOMES FOR OLDER ADULTS WITH HIGH CARE NEEDS

**DOI:** 10.1093/geroni/igae098.0073

**Published:** 2024-12-31

**Authors:** Jason Falvey, Amit Kumar

**Affiliations:** Chair; Discussant

## Abstract

Over the past decade, the CMS has introduced numerous value-based policy initiatives. While these changes aim to reduce cost of care, they often have disparate effects on patient care and clinical outcomes particularly among minority communities and older adults with high-needs. The first presentation explores the disproportionate impacts of Medicare Advantage (MA) penetration among skilled nursing facility (SNF) users across various racial and ethnic identities, shedding light on the disparities perpetuated within the healthcare system. Another will then examine the ramifications of the Patient-Driven Groupings Model (PDGM) on older adults with dementia receiving home care, scrutinizing the changes in care and potential impacts on outcomes associated with this shift. Following this, the discussion shifts to the inequitable access to adult day services in urban areas, particularly highlighting the barriers faced by many older adults seeking non-institutional long-term care options that are growing in number around the country. Last, the symposium will discuss findings from qualitative work exploring how neighborhood disadvantage affects community mobility and successful aging in place among older adults recovering from lower extremity surgery, and the structural inequities experienced by this population with changes to home health care benefits. We will specifically highlight factors such as community cohesion and exposures to violent crime as key deterrents for recovery among older adults living with new mobility disability. Overall, this symposium will stimulate current debate on merits and demerits of various federal policies and growth of MA plans for older adults with high-needs.

